# WRKY45 is a negative regulator of *Botrytis cinerea* resistance through the JA/ET signaling pathway in *Arabidopsis*

**DOI:** 10.3389/fpls.2025.1724180

**Published:** 2025-12-19

**Authors:** Huifang Ran, Zunyi Yuan, Zhihao Chen, Yongxin Mao, Shiwei Bao, Yuyu Chen, Mo Chen, Haiyan Zhang, Wenfeng Gong

**Affiliations:** 1 College of Plant Science, Xizang Agricultural and Animal Husbandry University, Nyingchi, Xizang Autonomous Region, China; 2 School of Resources and Environmental Engineering, Moutai Institute, Renhuai, Guizhou, China; 3 School of Brewing Engineering, Moutai Institute, Renhuai, Guizhou, China; 4 College of Forestry and Grassland, Xizang Agricultural and Animal Husbandry University, Nyingchi, Xizang Autonomous Region, China

**Keywords:** *Arabidopsis*, *Botrytis cinerea*, defense response, molecular mechanism, WRKY45

## Abstract

*Botrytis cinerea* is a necrotrophic fungal pathogen that causes significant crop damage, yet the molecular mechanisms underlying plant defense remain incompletely understood. Here, we identify WRKY45 as a negative regulator of *Arabidopsis* resistance to *B. cinerea* through the suppression of JA/ET-mediated defense signaling. Our results show that *WRKY45* expression was induced by *B. cinerea* infection, peaking 48 hours post-inoculation. Loss of *WRKY45* function enhanced resistance, while *WRKY45* overexpression increased susceptibility and cellular damage, as indicated by elevated electrolyte leakage, higher malondialdehyde levels, and reduced chlorophyll content. RNA-seq analysis identified 1,850 differentially expressed genes in *wrky45* mutants, with strong enrichment of JA/ET-responsive pathways. Defense-related genes, including *ORA59*, *PDF1.2*, *ERF104*, and *ERF1*, were markedly upregulated in *wrky45* but suppressed in overexpression lines, as confirmed by qRT-PCR. Electrophoretic mobility shift assays and dual-luciferase assays demonstrated that WRKY45 directly binds to the *ORA59* promoter inhibiting its transcription, and represses the expression of *PDF1.2*, *ERF104*, and *ERF1*. Together, these results show that WRKY45 functions as a negative regulator by suppressing the expression of JA/ET-mediated defense genes, thereby modulating plant resistance to *B. cinerea*.

## 1 Introduction

Necrotrophic fungal pathogens are a major threat to global agriculture by inducing host cell death and colonizing necrotic tissues as nutrient sources. Their airborne dissemination further accelerates disease spread. Among them, *B. cinerea* infects over 200 plant species and causes severe pre- and post-harvest losses worldwide (Dean et al., 2012; van Kan, 2006; Bi et al., 2021). Although jasmonic acid (JA), ethylene (ET), and salicylic acid (SA) signaling pathways are known to orchestrate plant immune responses (Glazebrook, 2005; AbuQamar et al., 2017; Song et al., 2022), the transcriptional regulatory frameworks that integrate these signals and orchestrate robust immunity against necrotrophs are still not fully understood.

In *Arabidopsis*, the JA/ET signaling module plays a pivotal role in resistance to *B. cinerea*. Core defense-associated genes, including *ORA59*, *PDF1.2*, *ERF104*, and *ERF1*, function as critical hubs within this pathway, integrating hormonal cues and activating antimicrobial responses. ORA59 functions as a central integrator of the JA and ET signaling pathways (Pré et al., 2008; Ju et al., 2017), whereas PDF1.2 encodes a plant defensin that serves as a hallmark of JA/ET-mediated defense (Penninckx et al., 1996; Cheng et al., 2018). ERF104 is an ET-responsive factor, and related studies indicate that it plays a crucial role in resisting non-adapted pathogens (Chen et al., 2013). ERF1 amplifies resistance by coordinating a broad spectrum of JA/ET-dependent defense programs (Lorenzo et al., 2003; Rasool et al., 2015). The activities of these JA/ET-responsive genes are strongly shaped by upstream transcription factors, highlighting the importance of transcriptional regulation in controlling immune outputs.

Among transcription factors, WRKY proteins have emerged as master regulators of plant immunity. They constitute one of the largest transcription factor families in plants and exert diverse functions by binding to W-box cis-elements in target promoters (Chen et al., 2012; Jiang et al., 2017; Zhu et al., 2022). WRKY transcription factors act as critical regulators of stress responses, functioning either as activators or repressors of defense genes to fine-tune plant immunity (Wang et al., 2025; Chen et al., 2025; Du et al., 2023). For example, WRKY33 is a well-characterized positive regulator of resistance to *B. cinerea*, enhancing JA/ET signaling, reactive oxygen species (ROS) homeostasis, and secondary metabolism (Birkenbihl et al., 2012; Liu et al., 2015). WRKY75 similarly promotes JA-mediated immunity (Chen et al., 2021). OsWRKY67 positively regulates plant disease resistance through the modulation of defense-related genes (Liu et al., 2018). contrast, several WRKYs—including SlWRKY3, PtrWRKY89, and VqWRKY52—function as negative regulators to prevent excessive immune activation (Luo et al., 2024; Jiang et al., 2016; Wang et al., 2017). These functional divergences highlight the context-dependent nature of WRKY function in plant immunity.

WRKY45 is a member of the WRKY transcription factor family and has been reported to participate in various physiological processes in *Arabidopsis*, such as responses to phosphorus starvation, salt stress, cadmium toxicity, and leaf senescence (Wang et al., 2014; Zhou et al., 2025; Li et al., 2023; Chen et al., 2017; Vatov et al., 2021; Barros et al., 2022). However, its role in plant immunity remains poorly understood, particularly in defense against necrotrophic pathogens. Considering the dual regulatory roles of WRKY transcription factors and the central importance of JA/ET-regulated defense genes such as *ORA59*, *PDF1.2*, *Thi2.1*, and *ERF1*, elucidating the function of *WRKY45* in *Arabidopsis* immunity against *B. cinerea* is critical.

In this study, we investigate the role of WRKY45 in modulating *Arabidopsis* resistance to *B. cinerea*. We specifically assess whether WRKY45 regulates key JA/ET-dependent defense genes and dissect the transcriptional mechanisms underlying its activity. Our findings demonstrate that WRKY45 functions as a negative regulator of plant immunity, thereby providing new insights into the transcriptional networks shaping host responses to necrotrophic pathogens and offering potential targets for enhance disease resistance in crops.

## 2 Materials and methods

### 2.1 Plant materials and growth conditions

The experimental materials included *Arabidopsis* (Columbia ecotype, *Col-0* wild type), as well as *wrky45* mutant lines (*WRKY45-RNAI-3* and *wrky45*) (Wang et al., 2014; Chen et al., 2017), and *WRKY45* overexpression lines (*35S:WRKY45–2* and *35S:WRKY45-6*). Seeds of *Arabidopsis* were surface-sterilized with 20% bleach solution for 15 minutes, and then sown on half-strength Murashige and Skoog (MS) solid medium (pH 5.8). After stratification at 4°C for 3 days, the plates were transferred to a greenhouse under a 16-hour light/8-hour dark photoperiod at 22°C. After 7 days of growth, seedlings were transplanted into moist soil and covered with plastic film, which was removed after 2–3 days to allow further growth. Seeds of *Nicotiana benthamiana* were grown in a green house at 25°C under a 16 h light/8 h dark photoperiod. In all experiments, Arabidopsis seedlings used for *B. cinerea* inoculation were consistently two weeks old. The *WRKY45* overexpression lines were constructed and maintained at the Key Laboratory of Sustainable Utilization of Tropical Plant Resources, Xishuangbanna Tropical Botanical Garden, Chinese Academy of Sciences. Verification of the *WRKY4*5 overexpression lines (*35S:WRKY45–2* and *35S:WRKY45-6*) is provided in **Supplementary Figure 1**.

### 2.2 Pathogen inoculation with *B. cinerea*

*B. cinerea* strain B05.10 was cultured on potato dextrose agar (PDA) at 22°C under a 12-hour light/12-hour dark photoperiod (Xiang et al., 2020). For whole-plant inoculation, *B. cinerea* conidial suspension was evenly sprayed onto the leaves of 2-week-old *Arabidopsis* seedlings, which were then maintained under short-day, high-humidity, and dark conditions to promote infection (Scholz et al., 2023). Samples were collected at 0 and 5 days post-inoculation (dpi) for physiological measurements, and disease symptoms were monitored daily. For detached leaf inoculation, *B. cinerea* conidia were suspended in SMB (40 g/L maltose, 10 g/L peptone) with 0.1% Tween 80, and the suspension was filtered through gauze to obtain a conidial solution. A 3 μL droplet of this suspension (5 × 10^5^; spores/mL) was applied to the abaxial surface of 14-day-old soil-grown *Arabidopsis* leaves, with 3 μL of SMB solution as a control. The leaves were placed on moist filter paper in Petri dishes and incubated under low light and high humidity at 22 °C for 3 days. Disease symptoms were assessed, and qualitative evaluation was performed using GUS staining.

### 2.3 RNA extraction and quantitative real-time PCR analysis

Seeds of *Arabidopsis* (different genotypes) were germinated on half-strength Murashige and Skoog solid medium, stratified at 4°C for 3 days and grown for 7 days before transferring to moist soil for 2 weeks. Seedlings were then inoculated with *B. cinerea*, and samples were harvested at various time points post-inoculation. Total RNA was extracted from *Arabidopsis* seedlings using the water-saturated phenol method. First-strand cDNA was synthesized by reverse transcription, followed by quantitative real-time PCR (qRT–PCR). Reverse transcription and qRT–PCR kits were obtained from Takara (Dalian, China), and PCR was performed on a QuantStudio 5 real-time PCR machine using SYBR Premix Ex Taq™ II. *ACTIN2* (AT3G18780) was used as internal control in qRT–PCR. Primer sequences used in qRT–PCR are listed in **Supplementary Table S1**.

### 2.4 RNA sequencing

Wild-type and *wrky45* mutant *Arabidopsis* lines, grown for two weeks, were inoculated with a suspension of *B. cinerea* spores and maintained under treatment conditions for five days. Samples were collected from both WT and *wrky45* mutant plants before and after inoculation. Total RNA was extracted from the seedlings using the water-saturated phenol method and subsequently sent to Kunming Hengyun Biotechnology Co., Ltd. for transcriptome sequencing. Three independent biological replicates were analyzed for each condition.

### 2.5 GUS staining

Histochemical GUS assays were performed as described previously (Chen et al., 2021). The GUS staining kit was purchased from Zhongke Ruitai Biotechnology Co., Ltd. (Beijing, China). Fourteen-day-old *WRKY45p:GU*S transgenic seedlings were detached and inoculated with *B. cinerea* for three days before GUS staining. Tissues were decolorized in 70% ethanol, with the solution replaced two to three times until the control tissues became completely white, and then photographed. Three independent biological replicates were processed under identical staining and imaging conditions to ensure reproducibility.

### 2.6 Electrolyte leakage assay

Electrolyte leakage was measured to assess membrane integrity in wild-type, *wrky45* mutant, and *WRKY45* overexpression seedlings at 0 and 5 dpi. Leaves were immersed in 10 mL of deionized water and gently shaken before measuring the initial conductivity (R_0_). Samples were then incubated at room temperature for 2 hours to allow passive ion leakage, and conductivity was measured again (R_1_). Subsequently, samples were boiled at 100°C for 20 minutes, cooled to room temperature, and final conductivity (R_2_) was measured. Electrolyte leakage was calculated as (R_1_ − R_0_)/(R_2_ − R_0_) × 100, following Bajji (Bajji et al., 2002). Three independent biological replicates were analyzed for each genotype.

### 2.7 Chlorophyll quantification

Fully expanded *Arabidopsis* leaves were collected, immediately frozen in liquid nitrogen, and ground to a fine powder. Photosynthetic pigments were extracted using ice-cold 80% (v/v) acetone, followed by centrifugation to remove debris. The Absorbance of the supernatant was measured at 663 nm and 645 nm using a UV–visible spectrophotometer. Chlorophyll *a*, chlorophyll *b*, and total chlorophyll contents were calculated using the Lichtenthaler equations for 80% acetone (Lichtenthaler, 1987). Three independent biological replicates were analyzed for each genotype.

### 2.8 Determination of malondialdehyde content

Malondialdehyde (MDA) content was determined using a thiobarbituric acid (TBA) method. Samples were collected from wild-type, mutant, and *WRKY45-*overexpressing *Arabidopsis* seedlings before and after *B. cinerea* infection. Approximately 1 g of fresh tissue was weighed, cut into small pieces and homogenized in 10 mL of 10% (w/v) trichloroacetic acid (TCA) using a mortar and pestle. The homogenate was centrifuged at 4,000 rpm for 10 min, and 6 mL of the supernatant was mixed with an equal volume of 0.6% (w/v) thiobarbituric acid solution. The mixture was incubated in a boiling water bath at 100 °C for 15 min, rapidly cooled to room temperature, and centrifuged for 1 min. The absorbance of the supernatant was measured at 450, 532, and 600 nm using a spectrophotometer, with 2 mL of distilled water as the blank control. MDA content was calculated according to the method described by Huang (Huang et al., 2016). Three independent biological replicates were analyzed for each genotype.

### 2.9 Electrophoretic mobility shift assays

To examine the potential interaction between WRKY45 and the W-box element, electrophoretic mobility shift assays (EMSA) were performed using a chemiluminescent EMSA kit (Beyotime Biotechnology, Shanghai, China) according to the manufacturer’s instructions. DNA fragments corresponding to the *ORA59* promoter region, containing either the wild-type W-box sequence or a mutated W-box motif, were synthesized and end-labeled with biotin. Purified recombinant GST-WRKY45 fusion protein was incubated with the labeled probes in binding buffer at room temperature for 20 minutes. For competition assays, a 50- to 100-fold molar excess of unlabeled probe was added in the reaction. The DNA–protein complexes were separated on 6% non-denaturing polyacrylamide gels and subsequently transferred to nylon membranes (Brunello et al., 2024). Biotin-labeled DNA was detected according to the kit protocol. The primers used for the EMSA assay are listed in **Supplementary Table 1**.

### 2.10 Transcriptional activity assays

The native promoters (about 2.5 kb) of *ORA59*, *PDF1.2*, *ERF1*, and *ERF104* were cloned into pGWB35 to generate the reporter constructs *ORA59p:LUC*, *PDF1.2p:LUC*, *ERF1p:LUC*, and *ERF104p:LUC* using Gateway technology (Invitrogen, USA). The reporter plasmids and effector constructs carrying 35S:WRKY45 were introduced into Agrobacterium tumefaciens strain EHA105 (pSoup). Agrobacterium cultures harboring the respective plasmids were grown, collected, and resuspended in infiltration buffer (10 mM MgCl_2_, 10 mM MES, and 200 μM acetosyringone) to an OD_600_ of 0.6 prior to infiltration (Kang et al., 2023). After infiltration, plants were maintained in a growth chamber under high humidity and low-light conditions for 72 hours. Before luminescence imaging, the infiltrated leaves were sprayed with fluorescein potassium salt solution (10 mL sterilized water + 10 μL 10% Triton X-100 + 0.008 g fluorescein potassium salt), incubated in the dark for 5 minutes, and imaged using a cooled CCD imaging system (Olympus, Japan) to capture LUC luminescence (Zhang et al., 2022). Each experiment was performed with at least three independent biological replicates. The primers used for the transcriptional activity assays are listed in **Supplementary Table 1**.

### 2.11 Statistical analysis

**Figure 1:**
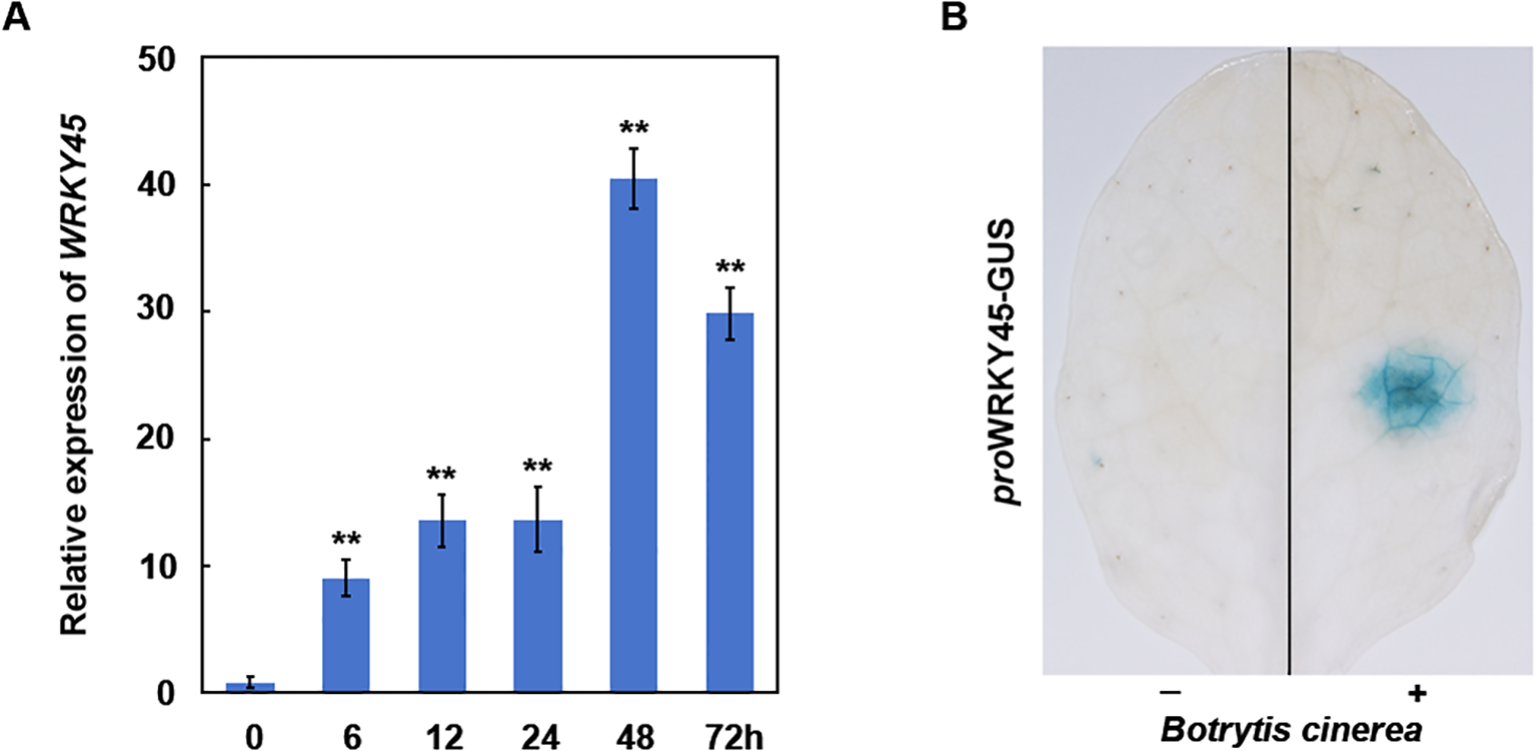
*WRKY45* expression is induced by *B. cinerea*. **(A)** Time-course analysis of *WRKY45* transcript accumulation in *Arabidopsis* following inoculation with (*B*) *cinerea*. Relative expression levels were quantified by qRT–PCR at 0, 6, 12, 24, 48, and 72 hours post-inoculation (hpi), using *ACTIN2* as the internal reference gene. Data are presented as the mean ± SD (n = 3 independent biological replicates). Statistical significance was determined using one-way ANOVA, and asterisks indicate significant differences compared to 0 h (**P < 0.01). **(B)** Histochemical GUS staining of *WRKY45p:GUS* transgenic plants after *B. cinerea* inoculation. Strong GUS activity was observed at the inoculation sites (+), compared to untreated control sites (–), confirming local induction of *WRKY45* expression by pathogen infection.

**Figure 2:**
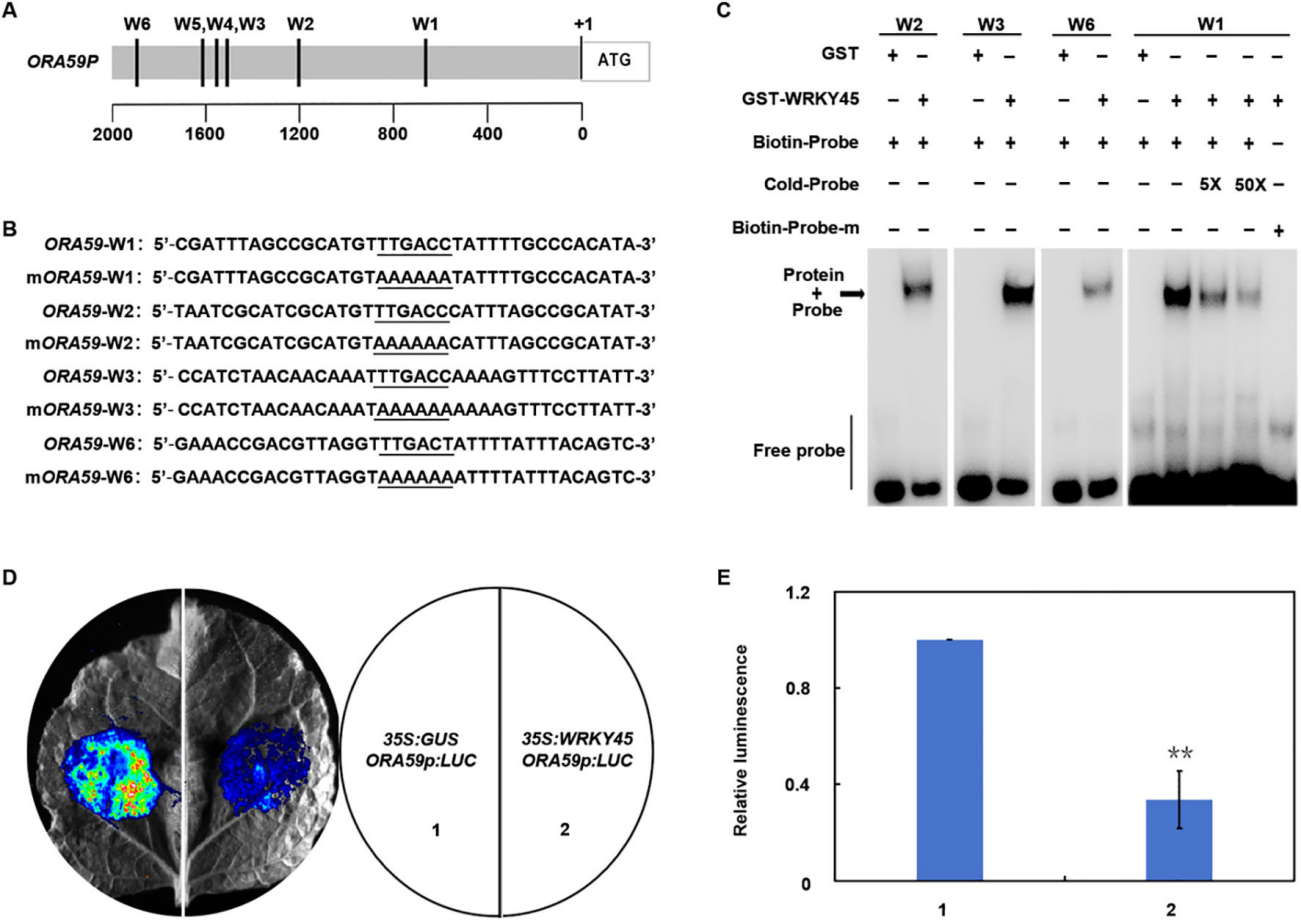
WRKY45 directly represses *ORA59* and downstream JA/ET-responsive defense genes. **(A)** Schematic representation of the *ORA59* promoter showing six putative W-box elements (W1–W6). **(B)** Nucleotide sequences of the W-box elements used in DNA-binding assays, with core motifs and mutated nucleotides underlined. **(C)** Electrophoretic mobility shift assay (EMSA) showing the interaction between GST–WRKY45 and W-box elements of the *ORA59* promoter. GST protein alone was used as negative control. “5×” and “50×” denote addition of fivefold and fiftyfold excess of unlabeled competitor probe, respectively; “+” and “−” denote presence or absence of competitor probes. **(D)** Transient dual-luciferase assays in *Nicotiana benthamiana* leaves. Representative luminescence images of co-infiltration with (1) *35S:GUS + ORA59p:LUC* (control) and (2) *35S:WRKY45* + *ORA59p:LUC* are shown. **(E)** Quantitative analysis of luciferase activity. Data are presented as the mean ± SD (n = 3 independent biological replicates). Statistical significance was determined using two-tailed Student’s t-test, and asterisks indicate significant differences (**P < 0.01).

**Figure 3:**
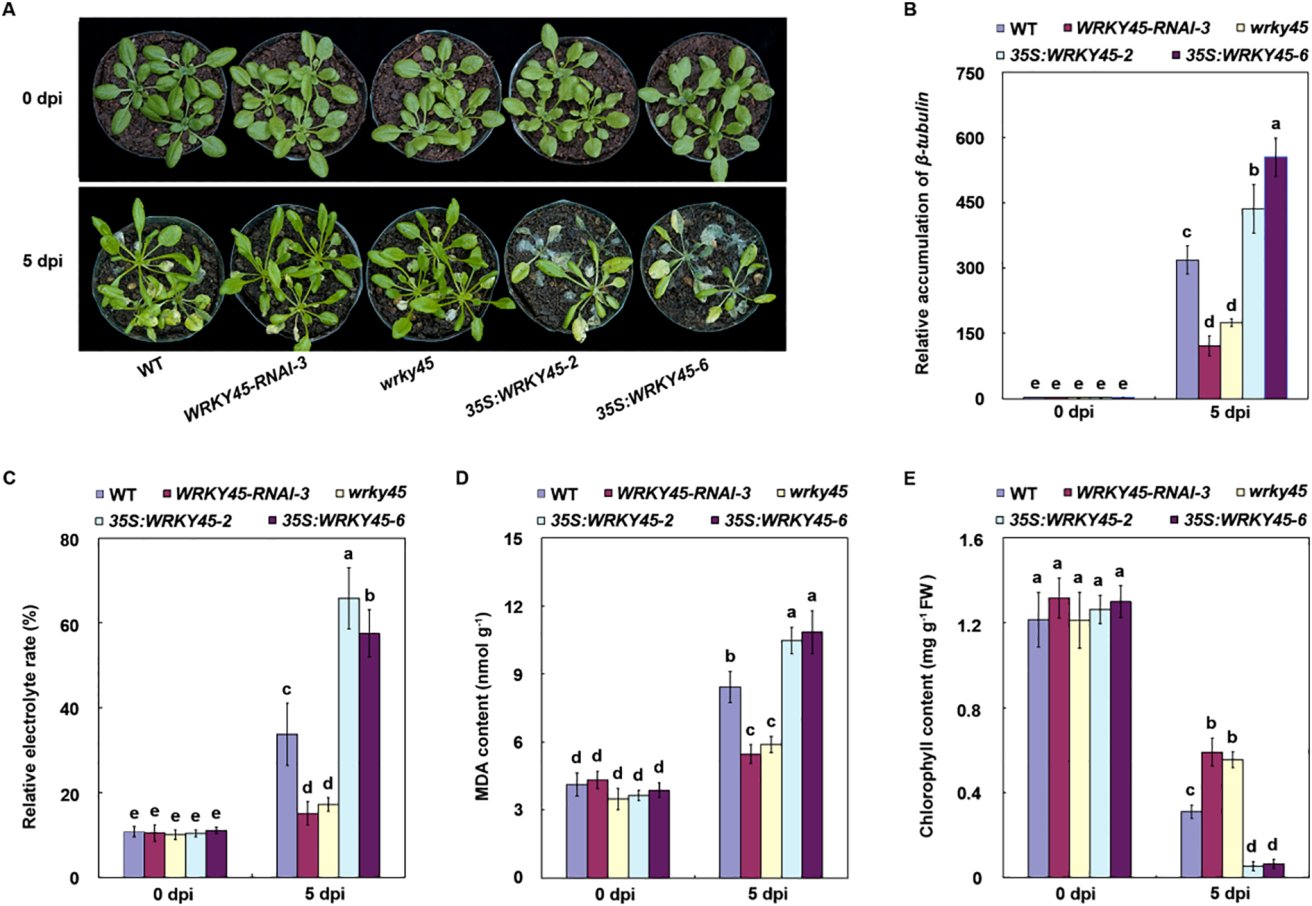
WRKY45 negatively regulates *Arabidopsis* resistance to *B. cinerea*. **(A)** Disease phenotypes of wild-type plants, *wrky45* mutants, *WRKY45−RNAi* lines, and *WRKY45* overexpression plants at 0 and 5 days post-inoculation (dpi) with *B. cinerea*. **(B)** Fungal biomass in infected leaves quantified by *β−tubulin* transcripts using qRT−PCR, with *ACTIN2* as the internal reference gene for normalization. **(C–E)** Electrolyte leakage, malondialdehyde content, and chlorophyll content at 0 and 5 dpi. Data are presented as the mean ± SD (n = 3 independent biological replicates). Statistical significance was determined using two-way ANOVA followed by Tukey’s test. Different letters above the bars indicate significant differences (*P *< 0.05).

All experiments were performed with at least three independent biological replicates, and data are presented as the mean ± standard deviation (SD). One-way ANOVA was used for **Figure 1A**, a two-tailed Student’s t-test for **Figure 2E**, and two-way ANOVA followed by Tukey’s test for **Figures 3**, **5**. Statistical significance was set at *P* < 0.05. Different lowercase letters indicate significant differences among multiple groups, while asterisks (* or **) denote significant differences in pairwise comparisons (*P < 0.05, **P < 0.01).

## 3 Results

### 3.1 WRKY45 is transcriptionally activated by *B. cinerea* infection

To investigate the role of WRKY4*5* in resistance to *B. cinerea*, wild-type seedlings were inoculated with a suspension of *B. cinerea* spores, and *WRKY45* transcript levels were assessed at various time points. qRT–PCR analysis revealed that *WRKY45* expression was rapidly and significantly upregulated within 6 hours post-inoculation, with transcript levels progressively increasing and peaking at approximately 48 h (**Figure 1A**), indicating that *WRKY45* expression is induced by *B. cinerea*. Subsequently, leaves of *WRKY45p:GUS* transgenic plants were inoculated with *B. cinerea*, and GUS staining was examined three days post-inoculation. A significantly stronger GUS signal was observed at the inoculation sites compared to the uninoculated regions (**Figure 1B**), further confirming the induction of *WRKY45* expression by *B. cinerea*. Collectively, these results demonstrate that WRKY45 is a pathogen-inducible transcription factor, potentially modulating defense responses to *B. cinerea*.

### 3.2 WRKY45 is a negative regulator of *Arabidopsis* responses to *B. cinerea*

To investigate the role of WRKY45 in *Arabidopsis* resistance to *B. cinerea*, we assessed the disease responses of wild-type, *wrky45* knockout mutants, *WRKY45-RNAi* lines, and *WRKY45* overexpression plants following *B. cinerea* inoculation. All lines exhibited comparable growth under normal conditions, with no discernible phenotypic differences. At 5 days post-inoculation, *wrky45* and RNAi plants exhibited significantly enhanced resistance compared with wild-type, whereas *35S:WRKY45* plants displayed pronounced susceptibility (**Figure 3A**). To quantify pathogen biomass, we examined the accumulation of *B. cinerea β-tubulin* transcripts at 0 and 5 dpi. At 0 dpi, *β-tubulin* levels were nearly undetectable across all genotypes. However, at 5 dpi, *β-tubulin* transcript abundance was markedly reduced in the *wrky45* and RNAi lines but substantially increased in the *WRKY45* overexpression plants (**Figure 3B**). These results consistently suggest that WRKY45 functions as a negative regulator of *Arabidopsis* resistance to *B. cinerea*.

To further evaluate the physiological consequences of WRKY45 activity, we measured membrane integrity, oxidative stress, and chlorophyll retention. The *wrky45* and RNAi plants exhibited significantly lower electrolyte leakage and malondialdehyde levels compared with WT, indicating reduced cellular damage, whereas *35S:WRKY45* lines showed exacerbated membrane injury and oxidative stress (**Figures 3C, D**). Moreover, chlorophyll content was better preserved in *wrky45* and RNAi plants but was strongly depleted in the *WRKY45* overexpression lines, correlating with their increased disease severity (**Figure 3E**). Collectively, these findings demonstrate that WRKY45 functions as a negative regulator of *Arabidopsis* resistance to *B. cinerea*.

### 3.3 RNA-seq analysis to identify the potential WRKY4*5-*involved pathway to control defense response

**Figure 4:**
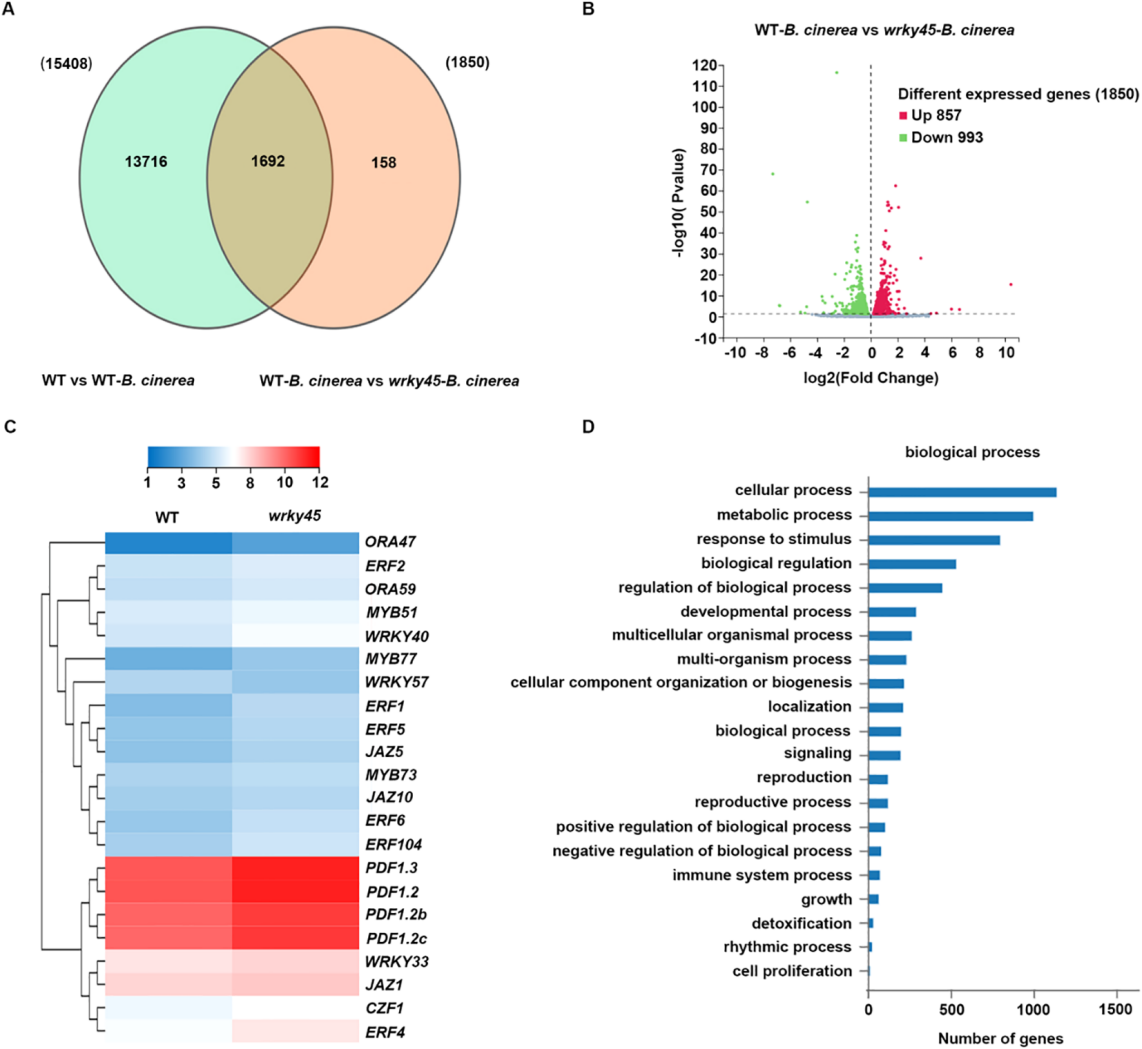
Transcriptomic analysis reveals WRKY4*5*-dependent regulation of defense-associated genes in response to *B. cinerea*. **(A)** Venn diagram showing the overlap of genes co-regulated by *B. cinerea* and *WRKY45* deficiency. The overlapping region represents genes co-regulated in the comparisons WT *vs*. WT–*B. cinerea* and WT–*B. cinerea vs*. *wrky45*–*B. cinerea*. **(B)** Volcano plot displaying differentially expressed genes (DEGs) between WT–*B. cinerea vs*. *wrky45*–*B. cinerea*. DEGs were defined using thresholds |log_2_ fold change| ≥ 1 and adjusted P value (Padj) < 0.05. The X-axis represents the fold change of the difference after conversion to log_2_, and the Y-axis represents the significance value after conversion to-log_10_. Red and green dots indicate up-regulated and down-regulated DEGs, respectively. **(C)** Hierarchical clustering of representative JA/ET-responsive, defense-related genes in the *wrky45* mutant following (*B*) *cinerea* infection. **(D)** Gene Ontology (GO) enrichment analysis of 1,850 DEGs in the *wrky45* mutant after (*B*) *cinerea* treatment, highlighting significant enrichment in biological processes related to plant immunity.

To elucidate the transcriptional basis of WRKY45-mediated defense, we performed RNA-seq analysis on wild-type and *wrky45* plants, with samples collected before and after inoculation with *B. cinerea*. A Venn diagram analysis revealed 15,408 differentially expressed genes (DEGs) in WT versus WT-*B. cinerea*, while 1,850 DEGs were identified in *wrky45* versus WT under infection conditions, with 1,692 genes shared between the two comparisons (**Figure 4A**). Among the 1,850 DEGs detected in the *wrky45* mutant, 857 were up-regulated and 993 were down-regulated (**Figure 4B**). Hierarchical clustering of representative defense-related genes showed that loss of *WRKY45* significantly enhanced the expression of several JA/ET marker genes, including *PDF1.2*, *PDF1.2b*, *PDF1.2c*, and *ORA59*, as well as stress-associated transcription factors such as *WRKY33*, *MYB77*, and multiple *ERF* genes (e.g., *ERF1*, *ERF4*, *ERF5*, *ERF6*) (**Figure 4C**). Notably, these genes are well-established regulators of immunity against necrotrophic pathogens, indicating that WRKY45 negatively modulates JA/ET-mediated defense transcriptional networks. Gene Ontology (GO) enrichment analysis further revealed that DEGs between WT and *wrky45* plants upon infection were predominantly enriched in biological processes associated with immunity, such as “response to stimulus,” “immune system process,” “detoxification,” and “regulation of biological process” (**Figure 4D**). Collectively, these results demonstrate that WRKY45 deficiency reprograms transcriptional responses toward enhanced activation of defense-related pathways, thereby strengthening resistance to *B. cinerea.*

### 3.4 WRKY45 represses the expression of JA/ET-dependent defense genes

**Figure 5:**
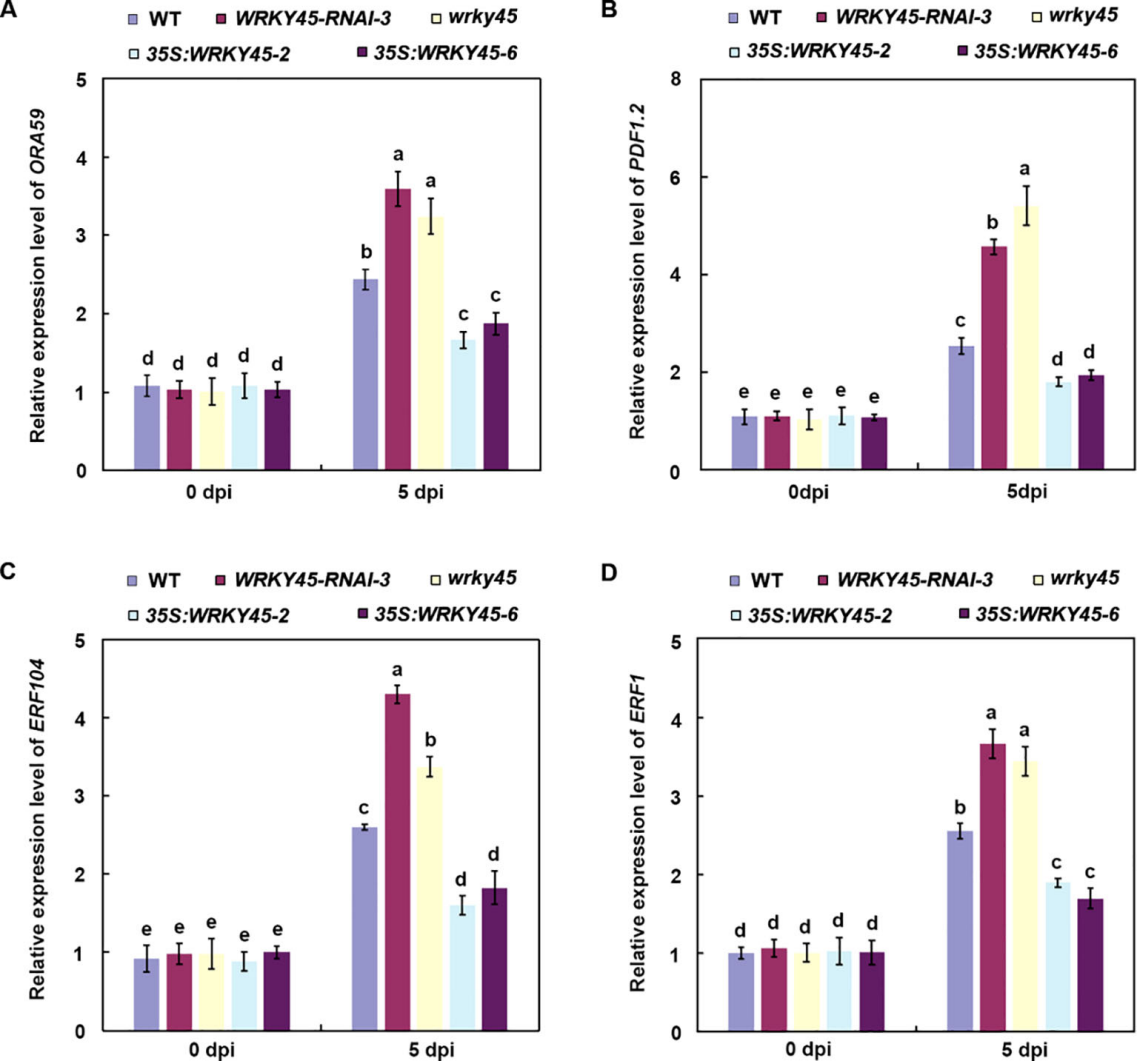
WRKY45 represses the expression of JA/ET-dependent defense genes. **(A–D)** Relative expression levels of *ORA59***(A)**, *PDF1.2***(B)**, *ERF104***(C)**, and *ERF1***(D)** were quantified by qRT–PCR in wild-type, *wrky45* knockout mutants, *WRKY45-RNAi* lines, and *WRKY45* overexpression plants at 0 and 5 days post-inoculation (dpi) with *B. cinerea*. using *ACTIN2* as the internal reference gene. Data are presented as the mean ± SD (n = 3 independent biological replicates). Statistical significance was determined using two-way ANOVA followed by Tukey’s test. Different letters above the bars indicate significant differences (*P* < 0.05).

To validate the transcriptional regulation of defense genes revealed by the RNA-seq analysis, we examined the expression levels of multiple JA/ET-dependent genes in different plant lines following *B. cinerea* infection. qRT–PCR analysis showed that the expression levels of *ORA59*, *PDF1.2*, *ERF104*, and *ERF1* in the *wrky45* mutant and *WRKY45-RNAi* plants were not significantly different from those in the wild type at 0 dpi, but were markedly upregulated at 5 dpi relative to the wild type (**Figures 5A–D**). By contrast, their transcript levels were significantly repressed in *WRKY45* overexpression lines, consistent with their enhanced susceptibility (**Figures 5A–D**). These findings, together with the RNA-seq results, demonstrate that WRKY45 functions as a negative regulator of JA/ET-mediated defense by repressing key downstream immune genes, thereby attenuating the host immune response against *B. cinerea*.

### 3.5 WRKY45 directly binds to the *ORA59* promoter and represses its transcriptional activity

To investigate whether WRKY45 directly regulates *ORA59*, we analyzed the *ORA59* promoter and identified several putative W-box elements (W1–W6) (**Figures 2A, B**). Electrophoretic mobility shift assays showed that recombinant GST–WRKY45 protein strongly bound to probes containing the W2, W3, and W6 elements, whereas while no binding signal was detected with the mutated W1 probe (**Figure 2C**). Competitive binding assays further demonstrated that an excess of unlabeled probes effectively blocked binding, confirming the specificity of these interactions (**Figure 2C**). To assess the functional relevance of this binding, transient dual-luciferase assays were performed in *Nicotiana benthamiana* leaves. Co-expression of WRKY45 with the *ORA59* promoter-driven LUC reporter significantly reduced luminescence compared to the control, indicating that WRKY45 suppresses *ORA59* transcriptional activity (**Figures 2D, E**). Additionally, transient assays using the promoters of *PDF1.2*, *ERF104*, and *ERF1* revealed that WRKY45 consistently repressed their transcriptional activity (**Supplementary Figure 2**). Taken together, these findings demonstrate that WRKY45 functions as a transcriptional repressor by directly binding to the *ORA59* promoter and suppressing JA/ET-dependent immune genes, thereby attenuating *Arabidopsis* resistance to *B. cinerea*.

## 4 Discussion

Gray mold, caused by the fungal pathogen *B. cinerea*, poses a significant threat to a wide range of crop species. WRKY transcription factors are crucial regulators of plant growth, development, and stress adaptation. In *Arabidopsis*, the functional characterization of individual WRKY members under both biotic and abiotic stresses has been key to uncovering the transcriptional networks involved in plant immunity. Here, we provide mechanistic insights into how WRKY45 modulates resistance to *B. cinerea* using *Arabidopsis* as a model.

Our phenotypic and physiological analyses show that *WRKY45* loss-of-function plants exhibit enhanced resistance to *B. cinerea*, while overexpression lines display increased susceptibility. These findings align with previous reports that several WRKY proteins act as immune repressors. For example, WRKY7, WRKY11, and WRKY17 suppress basal defense by downregulating JA and SA associated signaling (Fuenzalida-Valdivia et al., 2024). Notably, the phenotypes of *WRKY45* mutants are similar to those of the *wrky70 wrky54* double mutant, which exhibits enhanced resistance to necrotrophs, including *B. cinerea*, and shows strong induction of JA/ET- and SA-responsive genes (Li et al., 2017). These similarities support the conclusion that WRKY45 is part of a broader group of WRKY repressors that maintain immune homeostasis. However, unlike these previously described WRKY proteins, WRKY45 directly targets ORA59, a central integrator of JA/ET signaling, suggesting a more specialized role at a critical signaling node.

Transcriptomic profiling further revealed that WRKY45 deficiency leads to the strong activation of hallmark JA/ET-responsive genes, including *ORA59*, *PDF1.2*, *ERF104*, and *ERF1*. This transcriptional profile contrasts with that of *wrky33* mutants, where WRKY33 primarily regulates camalexin biosynthesis and ROS homeostasis (Birkenbihl et al., 2012; Liu et al., 2015). In contrast, this pattern more closely resembles the WRKY57-mediated repression of *B. cinerea* resistance described by Jiang and Yu, which acts through the JA signaling pathway (Jiang and Yu, 2016). These distinctions underscore the functional diversification within the WRKY family, where distinct members either activate or repress defense pathways depending on physiological and environmental contexts.

Mechanistically, we show that WRKY45 directly binds multiple W-box motifs in the *ORA59* promoter and suppresses its transcriptional activity. Since ORA59 integrates JA and ET signals, its repression by WRKY45 provides a highly efficient mechanism for modulating downstream immune activation. This direct suppression at the promoter level distinguishes WRKY45 from WRKY11 and WRKY17, which modulate defense indirectly through JA signaling cascades (Journot-Catalino et al., 2006). Moreover, WRKY45’s ability to repress the promoters of *PDF1.2*, *ERF104*, and *ERF1* suggests that it may coordinate a broader transcriptional repression module that limits JA/ET-mediated immunity.

Despite these findings, several important questions remain. Whether WRKY45 is involved in the hormonal interplay between JA, SA, and ET is still unclear. Additionally, *B. cinerea* infection is characterized by dynamic shifts from biotrophic-like to necrotrophic growth (Reboledo et al., 2020). High-resolution temporal transcriptomics, chromatin accessibility profiling, or single-cell RNA-seq could provide further insights into how WRKY45 activity changes during these distinct infection phases.

In conclusion, our results establish WRKY45 as a transcriptional repressor that attenuates JA/ET-mediated defense against *B. cinerea*. By directly suppressing *ORA59* and downstream immune genes, WRKY45 fine-tunes host defense responses. These findings enhance our understanding of WRKY-mediated transcriptional repression and open new avenues for translational research to enhance disease resistance in crops.

## 5 Conclusion

In this study, we identified WRKY45 as a transcriptional repressor that negatively regulates *Arabidopsis* defense against *B. cinerea* by suppressing JA/ET-mediated immune signaling. Loss of WRKY45 function enhanced resistance, reduced oxidative damage, and better preserved cellular integrity under pathogen challenge, whereas overexpression of *WRKY45* increased susceptibility to *B. cinerea*. Mechanistically, WRKY45 directly binds to W-box motifs in the ORA59 promoter inhibits its transcriptional activation, thereby attenuating the expression of downstream defense genes such as *PDF1.2*, *ERF104*, and *ERF1*. These findings not only enhance our understanding of the complex transcriptional networks governing plant immunity but also highlight the dual, context-dependent roles of WRKY transcription factors in fine-tuning stress responses. Given its conserved regulatory motifs, WRKY45 may serve as a promising target for molecular breeding and genome editing strategies aimed at enhancing crop tolerance to necrotrophic fungal pathogens.

## Data Availability

The original contributions presented in the study are publicly available. This data can be found here: https://www.ncbi.nlm.nih.gov/bioproject/PRJNA1390436.
