# Supplementary material for: WRKY45 is a negative regulator of Botrytis cinerea resistance through the JA/ET signaling pathway in Arabidopsis
Source: Front Plant Sci. 2025 Dec 19;16:1724180. doi: 10.3389/fpls.2025.1724180 (PMC12757795; doi:10.3389/fpls.2025.1724180)
Supplement: Supplementary file 1 [file DataSheet1.docx]

Supplementary Material

# Supplementary Data

# Supplementary Figures and Tables

## Supplementary Figures


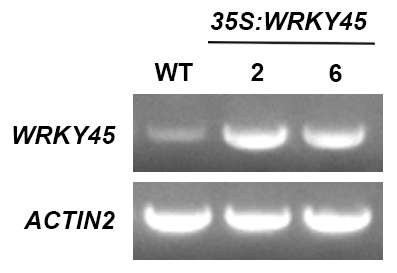


**Supplementary Figure 1.** Semi-quantitative RT-PCR analysis of *WRKY45* expression in two-week-old *Arabidopsis* wild type, *35S:WRKY45-2*, and *35S:WRKY45-6* plants, using *ACTIN2* as the internal reference gene.


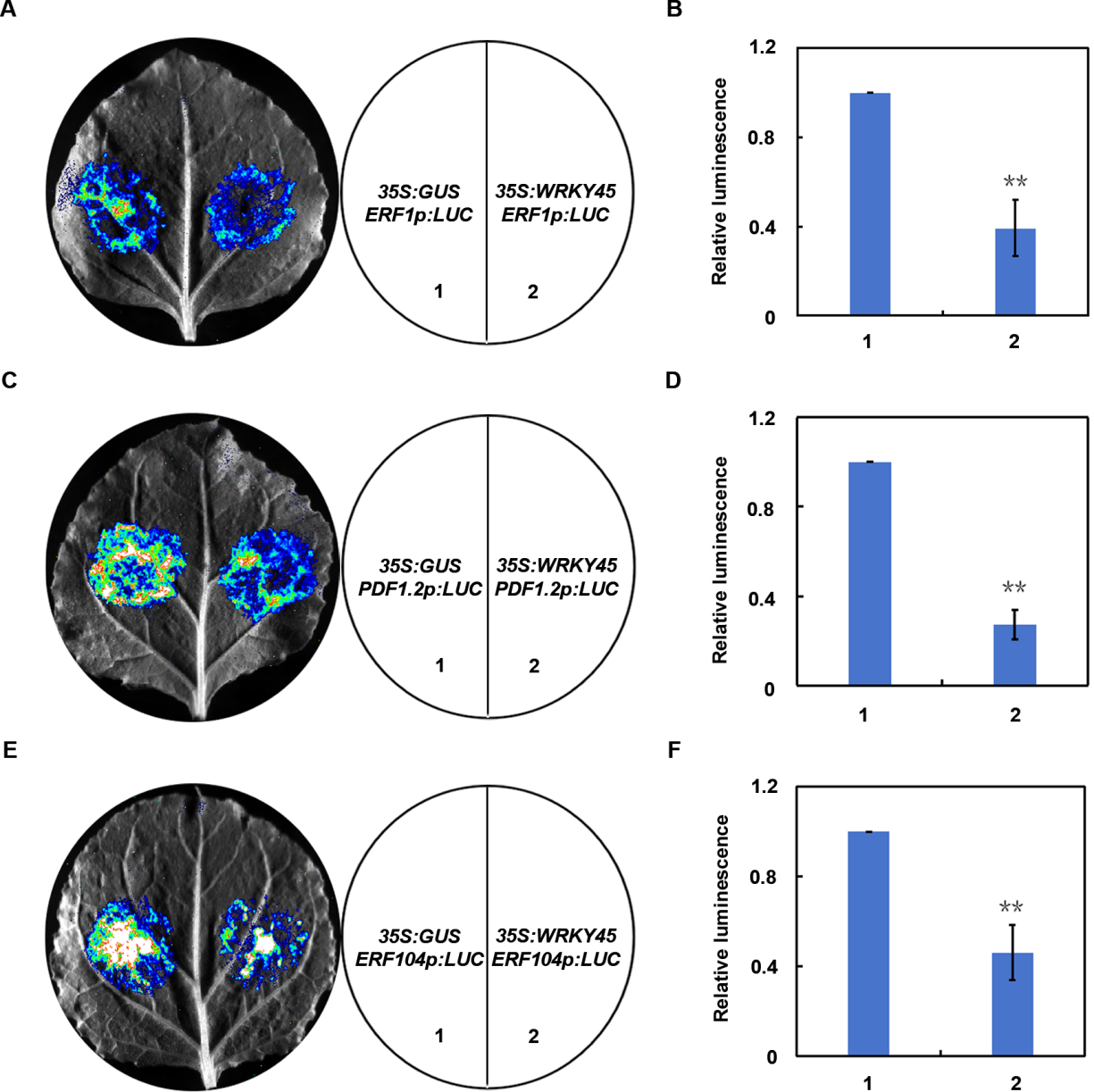


**Supplementary Figure 2.** WRKY45 exhibits transcriptional inhibitory effects on *PDF1.2*, *Thi2.1*, and *ERF1*. (A–B) Transient dual-luciferase assay in Nicotiana benthamiana leaves showing fluorescence images and quantitative analysis of luminescence intensity following co-transformation with ***35S:GUS* + *ERF1p:LUC*** or ***35S:WRKY45* + *ERF1p:LUC***. "1" represents ***35S:GUS* + *ERF1p:LUC*** (control), while "2" represents ***35S:WRKY45* + *ERF1p:LU*C**. (C–D) Fluorescence images and quantitative luminescence analysis for co-transformation with ***35S:GUS* + *PDF1.2p:LUC*** or ***35S:WRKY45* + *PDF1.2p:LUC***. (E–F) Fluorescence images and quantitative luminescence analysis for co-transformation with ***35S:GUS* + *Thi2.1p:LUC*** or ***35S:WRKY45* + *Thi2.1p:LUC***. In all assays, WRKY45 significantly repressed the transcriptional activity of the tested promoters compared with the control. Data represent mean ± SD from three independent biological replicates. **Asterisks denote statistical significance (**P < 0.01, Student’s t-test).

## Supplementary **Table**

**Supplement Table 1.** Primers used in this study.

| **Use** | **Gene name** | | **Primers (5’->3’)** |
| --- | --- | --- | --- |
| EMSA | | *ORA59* | *ORA59*-W1-F:  CGATTTAGCCGCATGTTTGACCTATTTTGCCCACATA  *ORA59*-W1-R:  TATGTGGGCAAAATAGGTCAAACATGCGGCTAAATCG  *ORA59*-W1-M-F: CGATTTAGCCGCATGTTTTTCCTATTTTGCCCACATA  *ORA59*-W1-M-R:  TATGTGGGCAAAATAGGAAAAACATGCGGCTAAATCG  *ORA59*-W2-F:  TAATCGCATCGCATGTTTGACCCATTTAGCCGCATAT  *ORA59*-W2-R:  ATATGCGGCTAAATGGGTCAAACATGCGATGCGATTA  *ORA59*-W3-F:  CCATCTAACAACAAATTTGACCAAAAGTTTCCTTATT  *ORA59*-W3-R:  AATAAGGAAACTTTTGGTCAAATTTGTTGTTAGATGG  *ORA59*-W6-F:  GAAACCGACGTTAGGTTTGACTATTTTATTTACAGTC  *ORA59*-W6-R:  GACTGTAAATAAAATAGTCAAACCTAACGTCGGTTTC |
| RT-qPCR | | *β-tubulin*  *ERF1*  *ORA59*  *PDF1.2*  *ERF104*  *ACTIN2* | *β-tubulin*-F: TCTTGAGAGCGGTGGTATC  *β-tubulin*-R: TTGCATACGATCGGAGATACCT  *ERF1*-F: TCTCTTCCCTTCAACGAGAACG  *ERF1*-R: GATTTGATCGGAAGGTCTTGACT  *ORA59*-F: AAAAGAAGAAGGAAAAGAAGCCAC  *ORA59*-R: GTGTCGAATGTCCCAAGCCA  *PDF1.2*-F: TCACCCTTATCTTCGCTGCTCT  *PDF1.2*-R: ATGATCCATGTTTGGCTCCTTC  *ERF104*-F : TTGGGACTTACGACACTGCC  *ERF104*-R : GGCGGAGAACCCTTATCTCG  *Actin2*-F: TGTGCCAATCTACGAGGGTTT  *Actin2*-R: TTTCCCGCTCTGCTGTTGT |
| Transcriptional activity assays | | *ORA59*   \| *PDF1.2* \| \| --- \|   *ERF1*  *ERF104*  *WRKY45* | \| *ORA59-F:* tgttacgttgagaaagaattcTGGTCATTTATATAATGAACGTATA \| \| --- \|   *ORA59-R:*  gtcttccattctagaggatccATTTCAATAGGTCCAATATGACA  *PDF1.2-F:*   \| tgttacgttgagaaagaattcAGCTATTTACGATTTAATATCTACTATA \| \| --- \|   *PDF1.2-R:*   \| gtcttccattctagaggatccTGGTAGTGGGTTAATCTTCTACT \| \| --- \|   *ERF1*-F:   \| tgttacgttgagaaagaattcGGTTACTTTTCTGATGTCAATATT \| \| --- \|   *ERF1*-R:   \| gtcttccattctagaggatccATGAGTATGTATATGCAAATGTCC \| \| --- \|   *ERF104*-F*：*   \| tgttacgttgagaaagaattcCGTTGATGAGTGGTCGCTTCT \| \| --- \|   *ERF104*-R*：*   \| gtcttccattctagaggatccCTTCACTCTACTTGATTGACTCTCTTG \| \| --- \|   *WRKY45*-F:   \| ATGGATCCATGGAGGATAGGAGGTGTGATGT \| \| --- \|   *WRKY45*-R:   \| ATTCTAGATCATTCCTTCAAGCAAAAGGG \| \| --- \| |
